# Reference values for the Modified Timed Up and Go Test in Saudi children aged 4–12 years old in Riyadh city: cross-sectional study

**DOI:** 10.1080/07853890.2021.1986638

**Published:** 2021-11-02

**Authors:** Nora Saleh Al-Toaimi, Afaf A. M. Shaheen, Maha Fahad Algabbani, Rehab F. M. Gawad

**Affiliations:** a Department of Rehabilitation Health Sciences, College of Applied Medical Sciences, King Saud University, Riyadh, Saudi Arabia; b Basic Science Department, Cairo University, Cairo, Egypt; c Physical Therapy Department, National Heart Institute, Giza, Egypt

**Keywords:** Timed Up and Go Test, reference values, functional mobility, dynamic balance, children, Saudi Arabia

## Abstract

**Purpose:**

The study aimed to report within-session reliability, estimate the reference values for the Modified Timed Up and Go (mTUG) test in typically developing (TD) Saudi children aged 4–12 years old, develop a reference equation for the estimated mTUG, and compare the measured mTUG in the present study with the predicted mTUG obtained from the previous regression equation.

**Methods:**

In this cross-sectional observational study, anthropometric measurements and mTUG test were investigated in 805 child. The association between the mTUG test and predictive variables was studied.

**Results:**

Average mTUG speed was 4.63 ± 0.68 s. Within-session reliability was excellent with intraclass correlation coefficient of 0.90. The test was significantly and negatively correlated with age, height, and weight (*r* = −0.66, *p* = .00), (*r* = −0.54, *p* = .01), and (*r* = −0.33, *p* = .01) respectively. According to the stepwise regression analysis, age and weight were the predictors and explained 47% of total variance of mTUG scores.

**Conclusion:**

This study provided the mTUG reference values that can be used clinically to evaluate functional mobility and dynamic balance in TD Saudi children aged 4–12 years. The mTUG scores can be predicted as a function of age and weight.

**KEY MESSAGES**
- Modified Timed Up and Go test used to assess the functional mobility and dynamic balance for children with or without developmental abnormalities.
- Availability of reference values according to age is helpful to compare the performance of children at same ages.

## Introduction

The Timed Up and Go (TUG) test assesses the functional mobility and dynamic balance, which are important requirements for daily activities. The test was categorised as activity by the International Classification of Functioning, Disability, and Health (ICF) [1]. It is applicable and affordable; moreover, it does not require specific training. Hence, it is widely used as a tool to assess the alteration of functional mobility in people of different ages with various medical conditions [2].

Initially, the TUG test was established in 1991 by Podsiadlo and Richardson [3] as a tool for assessment of older adults based on the original adaptation called Get-up and Go which was suggested by Mathias et al. in 1986 [4]. The duration of the test is calculated in seconds when the person stands up from a chair and walks three metres, turns around, walks back to the chair, and sits on it once more [3]. The factors that may impact the outcomes of the test must be considered when performing it. These factors are age, sex, height, weight, and body mass index (BMI) [5].

Over the past years, the TUG test was modified to be utilised with children. In the modified TUG (mTUG) test, children are asked to touch a target on a wall. To make the test more understandable to the children, the instructions were repeated whenever they needed [6]. Verbal encouragements can be given to children by the investigator, but they should be walking spontaneously. The chair used in the mTUG test should be carefully chosen, so the child’s hips and knees are flexed 90° [5]. The mTUG test is valid and reliable for assessing functional mobility among children aged from 3 to 14 age old [5]. Moreover, it was effectively applied on typically developing (TD) children and children with medical conditions such as spina bifida, traumatic brain injury, cerebral palsy, and Down syndrome [6–10]. Currently, the mTUG test is broadly utilised as a screening instrument for TD children [11]. The minimal equipment and space needed to apply the test make it an attractive and simple tool for school-based therapists. However, current age-based reference data are needed.

Earlier studies assessed the TD children in Pakistan, Australia, United States, Belgium, Brazil, and Hong Kong to obtain the reference values for TUG test and investigate the impact of probable predictive factors in exact populations [2,6,10–14]. Only one of those studies was conducted by Nicolini-Panisson and Donadio, 2013 to establish a predictive equation for Brazilian children aged 3–18 years. Since ethnicity is one of the strongest predictors for TUG scores, this equation is not valid to predict the TUG scores in other populations [2,10,15]. Although the TUG test is an important clinical assessment test, there is a lack of studies regarding the estimation of reference values and the impact of probable predictive factors on the mTUG test for TD Saudi children. Given this gap in the literature, the current study was conducted to report within-session reliability, estimate the reference values for the Modified Timed Up and Go (mTUG) test in typically developing (TD) Saudi children aged 4–12 years old, develop a reference equation for the estimated mTUG, and compare the measured mTUG in the present study with the predicted mTUG obtained from the previous regression equation.

## Methods

### Participants

A total of 805 participants were included in this study. They were divided according to the chronological age into 8 groups in 1-year increments [16].

The participants were included in this study if they were TD Saudi children aged 4–12 years, able to follow the instructions [11], did not have orthopaedic surgeries or fracture in the last 6 months [10,13,14]. On the other hand, they were excluded if they are using assistive aids (except glasses) such as orthosis and/or cochlear implants [2], complain of cardiorespiratory/neuromuscular disorders or musculoskeletal injuries [10,13,14], with severe visual or hearing impairment [2], and if there is any intellectual disability [10,11].

### Sample size

The calculation of sample size followed the steps. (1) Using the “rule of thumb method” (*n* ≥ 104 + *k*) [17] in which *n* = sample size and *k* = the number of independent variables [age, sex, height, weight, and BMI percentile] the sample size was 104 + 5 = 109. The expected withdrawal rate was 30% [18] therefore, at least 140 participants were required. (2) As suggested for stepwise regression analysis, the proper sample size must to be ≥200 participants [19]. (3) To have an excellent sample, size the number of participants in each sub-group must be more than 30 or 40 participants per sex [20]. According to these steps 891 participants were recruited.

### Ethical considerations

The ethical approvals were obtained from the Institutional Review Board (IRB), King Saud University (KSU), ethics number (CAMS No. E-19-3876), and by the Ministry of Education, ethics number (No. 5821).

### Design and setting

In this cross-sectional observational study, the TD Saudi children were recruited from Riyadh, Saudi Arabia (SA) using the stratified random sampling method from 10 schools and 5 kindergartens representing the five regions of Riyadh (centre, north, south, west, and east). The schools should be either private or governmental to have a varied and citywide representative sample. The data were collected from September to November 2019.

### Procedures

Prior to participants enrolment, parents/legal guardians were requested to sign a consent form that clarified the objectives and procedures of the research. They were requested to fill in a screening sheet with demographic information such as date of birth, sex, and school grade. Meanwhile, the sheet included general health questions such as if children have medical issues, balance abnormality, recent musculoskeletal injury, or abnormal hearing or vision to detect any exclusion criteria. More details were obtained from the parents by telephone if needed. In addition, face to face evaluation were carried out with all participants to confirm their eligibility for the study.

#### Anthropometric measurements

The weight in kilogram (kg) and the height in centimetre (cm) were measured using a calibrated weight and height scale (Cardinal Detecto ProDoc Series Physician Digital Scale). The participants were asked to remove their shoes, stand with minimum clothing in erect position. The calculation of BMI percentile for each participant was completed as stated by the Centres of Disease Control Prevention with the following categories: obese (≥95th percentile), over weight (85th to <95th percentile), healthy weight (5th to <85th percentile), and underweight (<5th percentile) [10].

#### Procedure of mTUG test

The mTUG test was performed following the protocol of Nicolini-Panisson and Donadio, 2013 [21]. This protocol would be the best protocol for the paediatric population because it has an excellent score according to COSMIN [20]. The administration of the mTUG test took around 5–10 min. The test was performed thrice in a quiet room. The participants could rest between the trials if needed. The mTUG test was explained once, and the researcher explained the instructions before starting the test and throughout the test if the participant is confused. The data were collected by one researcher.

All participants performed the test individually. The participant was inquired to sit on the chair, stand, walk 3-m distance as quick as possible, touch a target on the wall after the therapist says “Go”, return to the chair, and sit down once more. Time started when the participant stood up from the chair and stopped when the participant’s bottom touched the chair. The three successful trials per participant were documented and the within-session reliability was investigated. The test was repeated if the participant ran. The shortest time of the three trials was taken as a final result [21].

### Statistical analysis

Data were analysed using the statistical package for social sciences (IBM SPSS version 21). Confidence interval 95% was assigned therefore *p*-value ≤.05 was considered. Data distribution was examined prior to analysis using Shapiro–Willk test. The data were statistically treated to show the mean and standard deviation (SD) of age, height, weight, BMI percentile, and mTUG duration for all participants. Categorical data were expressed as frequency and percentage. Independent samples *t*-test was calculated to compare the anthropometric characteristics between the sexes.

Within-session reliability was examined using 2-way mixed-effects intraclass-correlation (ICC) model in which values greater than 0.90 indicate excellent reproducibility [22]. The two-way ANOVA with two factors (age and sex) was used to test the effect of age, sex, and the interaction effect of age and sex on mTUG scores. Post hoc analysis (Tukey HSD test) was utilised to determine the difference in every pair-wise condition. Pearson correlation coefficient (r) was calculated to test the correlation between the mTUG and independent variables (age, height, weight, and BMI percentile). Eta (*η*) was utilised to investigate the correlation between mTUG and sex. Linear regression model was utilised to assess the independent data explained the mTUG variance. The process of adding the independent factors to the model at each step was continued until no additional significant factors could be added. Variables entered and removed from the model depended on whether *p* > .05. The collinearity between the multivariate was detected by variance inflation factors at a cut-off point of 10 [23]. The measured mTUG was compared with the times predicted from the Brazilian equation [21] using Bland and Altman plot.

## Results

Out of 1500 consent forms and screening sheets received by parents/guardians, 891 participants were evaluated for this study with a response rate of 59.4%. Eighty-six children were excluded because of musculoskeletal injuries (*n* = 7), cochlear implant (*n* = 5), absent on the day of examination (*n* = 35) or refuse to participate in the study (*n* = 18). Totally, 805 children (373 boys and 432 girls) were eligible and completed the study.

### Demographic, anthropometric characteristics

All the data were normally distributed and homogenous. Table 1 demonstrates the anthropometrical attributes of the participants. The participants’ ages ranged from 4–12 years. The mean of age was 8.33 ± 2.30 year, and the mean of BMI percentile was 57.68 ± 34.03 (range, 1st to 99th percentile). Generally, there were no significant differences between boys and girls in respect to weight, height, and BMI percentile in most age groups (*p* > .05). For the entire sample, boys were significantly taller than girls (129.47 cm vs. 126.57 cm, *p* = .01) with no significant differences between boys and girls in all age groups except in 6 to <7 and 8 to <9 years (*p* < .05) in favour to boys. On the other hand, girls were significantly taller and heavier than boys in the age group 11–12 years (*p* < .05). The sample was classified as 8.4% underweight, 59.5% healthy weight, 13.5% overweight and 18.9% obese.

**Table 1.** Anthropometric characteristics of the participants.

Boys									Girls							
Age (years)	*n* (%)	Weight (Kg)	Height (cm)	BMI (%)	Categories of BMI (%)				*n* (%)	Weight (Kg)	Height (cm)	BMI (%)	Categories of BMI (%)			
					UW	HW	OW	O					UW	HW	OW	O
4 to <5	38 (10.2)	17.85 ± 2.22	107.76 ± 4.47	40.16 ± 30.8	6 (15.8)	27 (71.1)	2 (5.3)	3 (7.9)	49 (11.3)	18.38 ± 2.92	106.61 ± 4.22	56.78*±33.51	3 (6.1)	30 (61.2)	7 (14.3)	9 (18.4)
5 to <6	35 (9.4)	20.42 ± 3.87	112.77 ± 5.45	50.49 ± 36.48	5 (14.4)	21 (60)	2 (5.7)	7 (20)	37 (8.6)	19.30 ± 4.12	111.22 ± 4.54	48.65 ± 31.06	3 (8.1)	29 (78.4)	3 (8.1)	2 (5.4)
6 to <7	44 (11.8)	22.02 ± 4.77	117.66*±4.80	44.41 ± 37.04	6 (13.6)	27 (61.4)	4 (9.1)	7 (15.9)	50 (11.6)	21.37 ± 4.52	115.04 ± 5.15	52.80 ± 34.04	3 (6)	33 (66)	8 (16)	6 (12)
7 to <8	31 (8.3)	26.4 ± 7.72	124.87 ± 7.80	53.42 ± 39.23	6 (19.4)	15 (48.4)	3 (9.7)	7 (22.6)	56 (13)	25.25 ± 6.51	122.20 ± 6.46	58.52 ± 30.89	3 (5.4)	38 (67.9)	7 (12.5)	8 (14.3)
8 to <9	44 (11.8)	30.23 ± 7.82	131.32**±7.12	59.25 ± 34.69	4 (9.1)	24 (54.5)	6 (13.6)	10 (22.7)	69 (16)	28.87 ± 7.46	127.04 ± 7.19	63.51 ± 30.66	2 (2.9)	45 (65.2)	8 (11.6)	14 (20.3)
9 to <10	47 (12.6)	30.4 ± 5.67	133.19 ± 5.76	54.78 ± 33.51	3 (6.4)	31 (66)	10 (21.3)	3 (6.4)	57 (13.2)	33.89 ± 9.81	132.51 ± 7.07	64.60 ± 33.54	2 (3.5)	30 (52.6)	10 (17.5)	15 (26.3)
10 to <11	77 (20.6)	41.17 ± 13.67	142.47 ± 7.98	64.24 ± 37.32	10 (13)	29 (21.3)	10 (13)	28 (36.4)	57 (13.2)	39.52 ± 10.88	141.04 ± 8.47	65.46 ± 31.81	3 (3.5)	31 (54.4)	11 (19.3)	12 (21.1)
11 to 12	57 (15.3)	39.69 ± 8.88	143.51 ± 6.51	60.58 ± 33.51	5 (8.8)	33 (57.9)	8 (14)	11 (19.3)	57 (13.2)	43.69*±12.11	147.12**±8.69	64.05 ± 30.38	4 (7)	36 (63.2)	10 (17.5)	7 (12.3)
Total	373 (46.34)	30.49 ± 11.95	129.47**±14.20	54.92 ± 35.92	45 (12.1)	207 (55.5)	45 (12.1)	76 (20.4)	432 (53.66)	29.55 ± 11.89	126.57 ± 14.75	60.06*±32.16	23 (5.3)	272 (63)	64 (16.9)	73 (16.9)

Variables were illustrated as mean ± standard deviation (SD) except the categories of body mass index (BMI) percentile (Perce.,) as frequency and percentage.

*n*: Number of participants; UW: underweight; HW: healthy weight, OW: overweight; O: Obesity.

*Significant difference between boys and girls. *p ˂ .05; **p < .01.

### Reproducibility and within-session reliability of mTUG test

The mean of time ± SD to perform the first trial was 4.63 ± 0.68 s, the second trial was 4.83 ± 0.76 s, and the third trial was 5.10 ± 0.83 s. Evaluating the reproducibility, the within-session reliability was excellent with ICC of 0.94.

### mTUG test score by age and sex

Average mTUG speed was 4.63 ± 0.68 s. The results of the two-way ANOVA showed a statistically high significant effect of age (*F*(7, 789) = 120.08, *p* = .00) and a low significant main effect of sex (*F*(1, 789) = 4.26, *p* = .04) on mTUG. Furthermore, significant age × sex interaction was noted (*F*(7, 789) = 2.89, *p* = .01). Age has greater influence on mTUG (*η*^2^ = 0.52) compared to sex (*η*^2^ =0.005) while the effect size of age × sex interaction was 0.025 (Table 2). The post hoc analysis results demonstrated that children older than 7–12 years were significantly faster than younger ones (*p* < .05).

**Table 2.** Two-way ANOVA for the influence of age and sex in mTUG.

Source of Variation	Type III sum of squares	Df	Mean square	*F*	Sig	Partial *η*^2^
Main effect of age	186.80	7	26.69	120.08	0.00	0.52
Main effect of sex	0.95	1	0.95	4.26	0.04	0.005
Interactive effect (age × sex)	4.50	7	0.64	2.89	0.01	0.025
Error	175.34	789	0.22			
Total	17661.74	805				
Corrected total	373.72	804				

df: degrees of freedom, *F*: *F*-value, Sig.: significant level (<0.05), *η*^2^: eta-squared.

**Figure 1.**
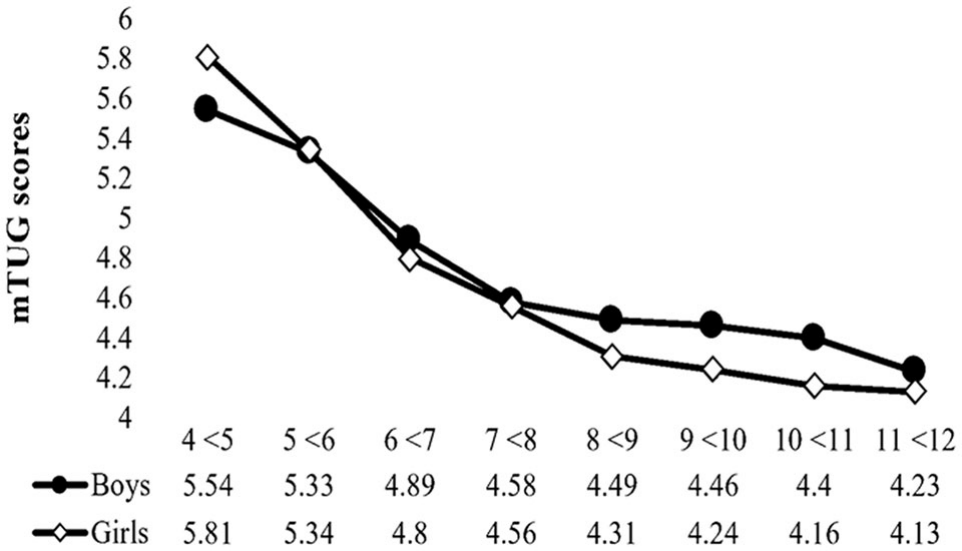
The mTUG test score by age and sex.

Figure 1 illustrated age × sex interaction; significant difference (P< 0.05) was found among children aged 4 to <5 years where boys were faster than girls by about 0.27 s. On the other hand, girls aged 9 to <10 and 10 to <11 years were significantly faster by 0.22 and 0.24 s respectively. In addition, girls aged 11 to <12 years were faster than boys by 0.10 s. These differences did not exceed the 2-s clinically important difference [21]. Generally, girls aged 6 years and above performed better than boys (Figure 1). The simple effect analysis revealed that the effect of age on mTUG performance was higher in girls compared to boys (partial *η*^2^ = 0.43 and 0.28 respectively).

### Factors affecting the performance of mTUG

There was a significant and negative correlation between the mTUG and age, height, and weight, (*r* = −0.66, *p* = .01), (*r* = −0.54, *p* = .01), and (*r* = −0.33, *p* = .01) respectively but not with BMI percentile (*r* = 0.05, *p* = .17). Based on Eta (*η*), the association between mTUG and sex was very weak (*η* = 0.05).

### Predicting factors of mTUG

The significantly associated factors (age, weight, and height) were involved in stepwise regression analysis to define the mTUG predictors, and develop the regression equations. The findings reported that age and weight were the foremost noteworthy and significant predictors. They explained 47% of total variance of mTUG scores (Table 3). The equation which includes both age and weight as predictors is as follow:

mTUG (Seconds)=6.27− [0.25×Age (years)]+[0.01×Weight (Kg)].

**Table 3.** Linear regression analysis for predicting mTUG.

Model	Independent variable	*R*	*R* ^2^	Unstandardised coefficient		Standardised coefficient	Sig.
				*B*	SE	*β*	
1	(Constant)			6.27	0.07		0.00
	Age	0.66	0.44	−0.20	0.01	−0.66	0.00
2	(Constant)			6.27	0.07		0.00
	Age			−0.25	0.01	−0.84	0.00
	Weight	0.69	0.47	0.01	0.00	0.25	0.00

SE: standard error; Sig.: significant; B: unstandardised regression coefficient; β: standardised coefficient.

### Comparison between the measured mTUG time in this study with the previous studies

**Figure 2.**
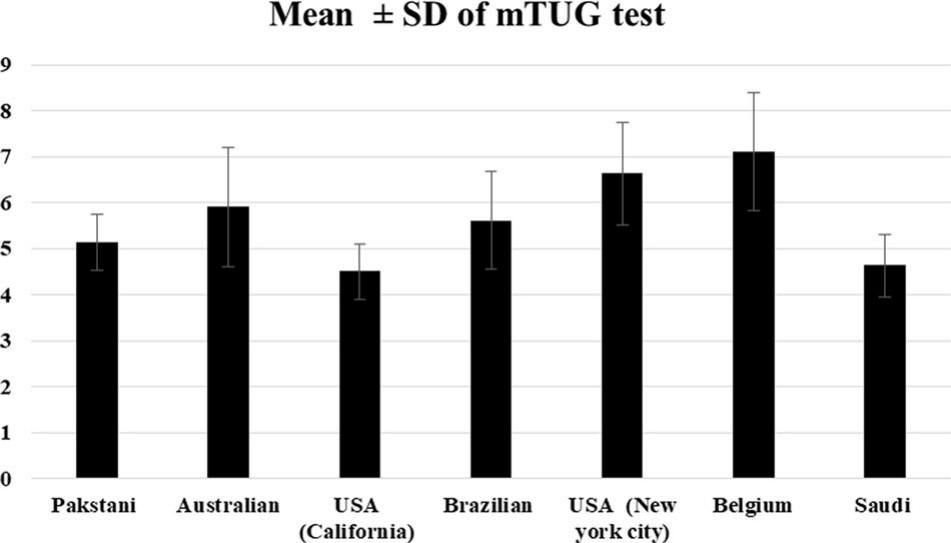
Comparison of mTUG scores measured in this study (Saudi) with previous studies.

The mean of mTUG for Saudi boys and girls over eight successive ages from 4 to 12 years compared to six previously published studies is illustrated in (Figure 2). The mean of mTUG is close to values of American children (California) [24] and lower than the values reported for Pakistani, American (New York City), Brazilian, Belgian, and Australian children [2,6,10–14].

**Figure 3.**
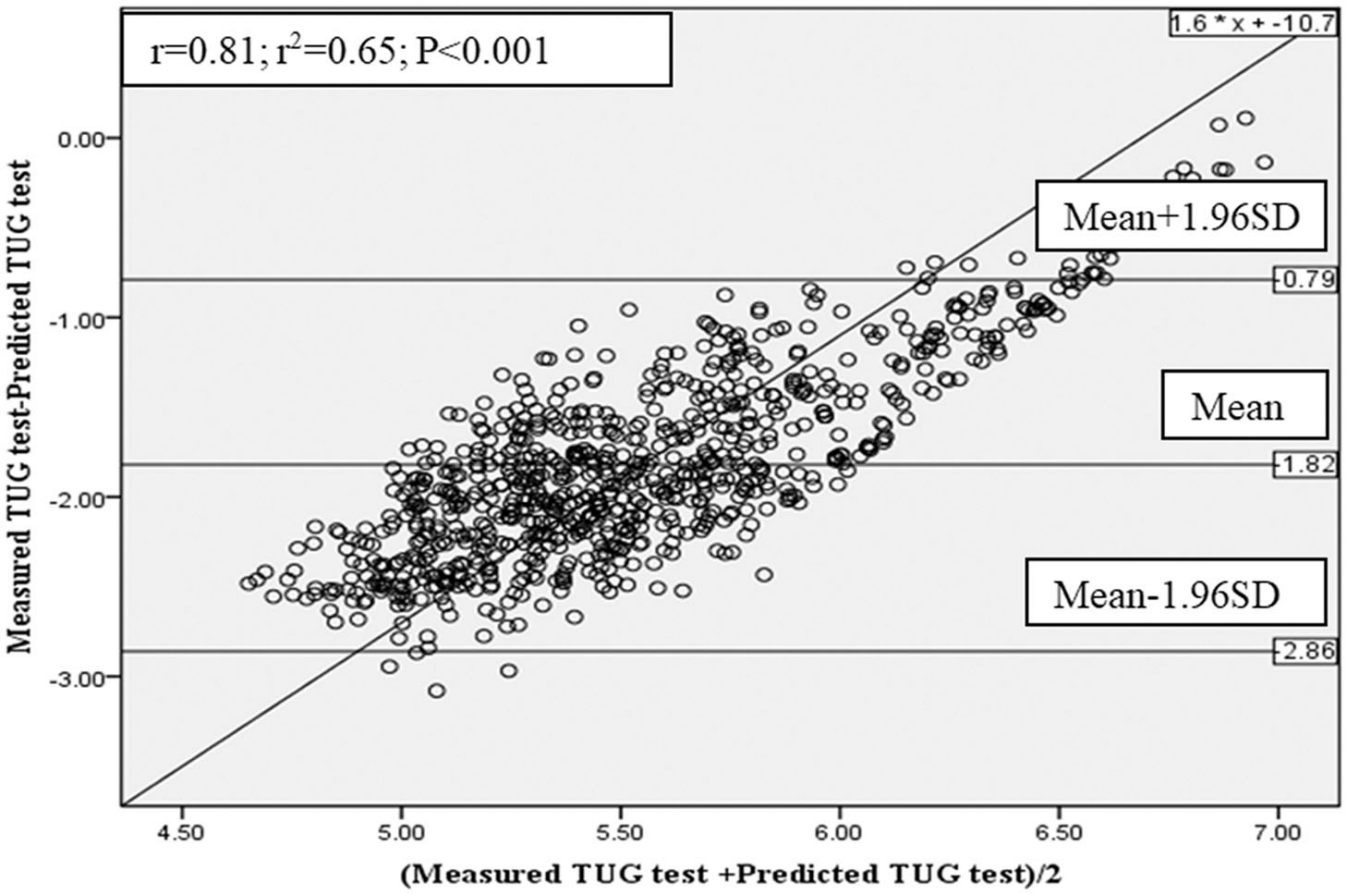
Bland and Altman plot, of measured and predicted mTUG determined from the reference equation of Brazilian children [10]. *r*^2^: determination-coefficient; *r*: correlation-coefficient; *p*: probability. Upper confidence interval value (CI) = Mean +1.96 SD. Lower confidence interval value (CI) = Mean − 1.96 SD.

Figure 3 illustrated the Bland and Altman comparison between the measured mTUG and mTUG predicted from the Brazilian regression equation [10]. There was a systematic bias between the measured and the predicted values from this equation. The correlation between mean difference (*Y*-axis) and mean value (*X*-axis) is significant (*r* = 0.81, *p* = .01), representing a proportional error of mTUG predicted with the corresponding reference equation. The mean ± SD of measured mTUG was also significantly lower by 1.82 ± 0.53 s than the Brazilian norms.

### Discussion

The aims of this study were to report within-session reliability, estimate the reference values for the mTUG test in a sample of TD Saudi children aged 4–12 years old, develop a reference equation for the estimated mTUG, and compare the measured mTUG in the present study with the predicted mTUG obtained from the previous regression equation. The results imply that the mean of mTUG test was 4.63 ± 0.68 s (4.67 ± 0.66 s for boys and 4.61 ± 0.70 s for girls). Based on regression analysis, the age and weight were the most significant and important predictors of the mTUG test. They were accounted for 47% of the total variance of mTUG test.

In line with the previous studies [2,6,10–14] the mean age was 8.33 ± 2.30 year. However, a Belgian study included only pre-school children [2] and the Brazilian study had a wider age range (3–18 years) [10]. In this study, the means of height and weight were 127.89 ± 1.46 cm and 29.98 ± 11.92 kg respectively. They are slightly less than those reported in Butz et al., study (131.6 ± 7.06 cm and 31.70 ± 7.78 kg) [24]. In agreement with Itzkowitz et al., 2016, most of our children have a healthy weight (59.5% vs 63.83%) [13].

Within-session reliability of mTUG test was excellent (ICC = 0.93, *p* < .05). This result is supported by a Nicolini-Panisson and Donadio study with ICC= 0.95 [10]. Furthermore, the ICC value was greater than the values reported by Lie et al. and Williams et al. studies (ICC= 0.74 and 0.80 − 0.89 respectively) [6,11].

By testing 805 children, the mTUG test scores can be used as reference values for Saudi children aged 4–12 years. Younger children demonstrated longer mTUG test time than older ones. The mTUG test is highly influenced by age [24–28]. The performance was significantly improved and the speed of mTUG test increased as age progressed. This finding is consistent with the previous studies [2,12,13,24].

Williams et al. conveyed that the improvement in the child’s performance over age may be attributed to the development in body strength and size [6]. Besides, the child’s balance improved with age; children aged 9 and above have better balance than younger children [29,30]. In consequence, the child’s speed increases as age progresses [21]. Futhermore, the child’s gait is developed towards a mature gait with increasing the age or because of the child’s cognitive functions such as concentration and attention may play a role [20]. Therefore, the score of mTUG test is sensitive to child’s age due to the maturation of balance during walking [20].

The results found that sex significantly affects the mTUG test performance. Besides, significant age × sex interaction was noted. Overall, girls aged 6 years and above performed better than boys. In agreement with these results, Izkowtiz et al. reported significant non clinically important differences between boys and girls [13] while the Pakistani study demonstrated that boys were significantly faster due to cultural issues, this difference could be due to wearing a chador [12]. Otherwise, the findings disagreed with the previous studies [6,10,24] which reported non-significant infeluence of sex on TUG test.

The influence of sex on mTUG performance can be explained according to Steindl et al.’s study [31]. Their results displayed those girls showed a greater rate of improvement in stability until the age of 11–12 years. Younger boys under the age of 10 years appeared to be less mindful and agitated. Riach and Hayes and Odenrick and Sandstedt noted that boys younger than 10 years swayed more than girls of the same age [32,33]. Moreover, Hirabayashi and Iwasaki reported that hyperactivity in young boys lead to delay their rate of maturation in posture control [34].

Sex differences in balance ability have been documented earlier. Girls demonstrated higher capability to control the direction of the centre of gravity than boys aged 9 and 10 years old which led to greater balance ability [25]. Moreover, girls showed earlier development of the integrated systems between their vestibular, ocular and proprioceptive senses that make smaller muscular reactions and results in more stable balance than boys [35,36]. More integration of these systems and righting response appears in boys between the age of 15 and 16 years [25].

Similar to the previous studies, our results showed that mTUG was significantly correlated with age, weight, and height but not with BMI [10,13].

The predictors for TUG time were examined by many studies. In Pakistani children, age accounted for 18% of the TUG test values [37]. In a sample of American children when permitting self-selected walking speed, age accounted for 24.3–49% of the TUG test values [13,24]. On the other hand, age and weight accounted for 25% of the variation of the TUG values in children from South Brazil, when they walked fast during the test [10]. In Belgium, ethnicity explained 28% of the variance in TUG time for pre-schoolers who walk fast [2]. Several authors reported that BMI [10,13] and body height [2,10,24] did not account for the variance in TUG time.

Interestingly, one study conducted by Nicolini-Panisson and Donadio in 2013 established a prediction equation to predict the value of TUG test in TD children [10]. This study offers an equation to predict mTUG value, where age and weight represent the predictive factors with a power of 47%. Only one study conducted by Nicolini-Panisson and Donadio established a prediction equation to predict the value of TUG test in TD children. They reported that age and weight accounted for 25% of the values of TUG in TD children [10].

Nowadays, TUG test has been used widely for children with or without development abnormality [21]. The most imprtant issue is the standardisation of the procedures, which has not been fully established, especially the type of verbal instructions [10,12] and turning-point markers [10,12,13]. The variations in the instructions and turning-point marker may affect the speed of the TUG in TD children with different sexes and ages. Recently, Bustam et al. reported that different protocols may influence the outcome [38].

Compared to the previous studies, the children in this study needed an average of 4.63 s to complete the test. In literature, the time needed for children to complete the TUG test was ranged from 4.5 s [24] to 7.19 s [2]. The variation in TUG time was due to several adjustments in the protocols for the paediatric population, such as walking speed, using varying verbal instructions, and different turning-point.

The difference in mTUG scores between this study and Belgian study (4.63 and 7.19 s respectively) can be attributed to turning-point (grasping and transporting task) which could slow the walking speed. In addition, they enrolled younger children aged 3–6 years [20]. Further, in this study the scores of the mTUG were comparable to the American study [24]. The turning-point was the same (touching a target on the wall), but the walking instructions were different.

Although this study and Nicolini-Panisson and Donadio’s study [10] followed the same protocol, the mTUG reference values varied. This may be a result of data presentation and how the age was reported. In this study, we reported the age chronologically (1-year increments) while the other study described the age as age band. Moreover, the mean of mTUG was lower than the values reported in Pakistani (5.2 s), Australian (5.9 s), and American (6.63 s) studies. In these studies, the slow performance could be explained by instruction given to the child to walk in normal speed [2,12,13,24] and the type of turning point (a tape placed on the floor) [13].

To the best of authors’ knowledge this is the first study conducted in SA. It was the first that used a randomised sample method with large sample size within each age group. Nevertheless, the study has some limitations. It did not include children over 12 years and was performed only in one city of SA. However, Riyadh is the first-largest city that attracts citizens from different provinces. Since data were gathered from different regions of the city, the results may be valid for other Saudi children too. However, further studies including different geographic regions are needed. Meanwhile, it is strongly recommended that the regression equation should be validated in other regions of SA. Further studies including children with different medical health conditions are recommended. Finally, other possible variables that may impact the mTUG performance for example the biological maturity, peripheral muscle strength, leg length, cognitive status, and psychological factors were not evaluated in this study. Thus, further studies to evaluate the effect of these factors on the values of mTUG are warranted.

## Conclusion and clinical implications

This study establishes the reference values of mTUG for TD Saudi children aged 4–12 years old, from the largest city of SA. Significant influences of age, sex, and age × sex interaction were found and significant negative correlations between mTUG speed and age, height, and weight were also reported. Age and weight represent 47% of the variation of the mTUG values which lead then to be the most significant predictors for the test. The variation in the methodology, data presentation, and characteristics of the sample between the current study and the previous studies could be the reasons for the inconsistence of the TUG test values.

The predicted mTUG values may be helpful for comparing individual children to age-matched norms. It helps in the evaluation of interventions for patients with impaired functional mobility and dynamic balance. School-based physical therapists can use the mTUG as part of their assessment to govern whether students are functioning slower than age-matched peers when moving between their seats and other classroom locations.

## Data Availability

The data that support the findings of this study are available on request from the corresponding author [M.A]. The data are not publicly available due to their containing information that could compromise the privacy of research participants.

## References

[1] Geyh S, Cieza A, Schouten J, et al. ICF core sets for stroke. J Rehabil Med. 2004;36:135–141. 10.1080/16501960410016776 15370761

[2] Verbecque E, Vereeck L, Boudewyns A, et al. A modified version of the timed up and go test for children who are preschoolers. Pediatr Phys Ther. 2016;28(4):409–415. 27661232 10.1097/PEP.0000000000000293

[3] Podsiadlo D, Richardson S. The timed “up & go”: a test of basic functional mobility for frail elderly persons. J Am Geriatr Soc. 1991;39(2):142–148. 1991946 10.1111/j.1532-5415.1991.tb01616.x

[4] Mathias S, Nayak U, Isaacs B. Balance in elderly patients: the “get-up and go” test. Arch Phys Med Rehabil. 1986;67(6):387–389. 3487300

[5] Verbecque E, Lobo Da Costa PH, Vereeck L, et al. Psychometric properties of functional balance tests in children: a literature review. Dev Med Child Neurol. 2015;57(6):521–529. 25495539 10.1111/dmcn.12657

[6] Williams EN, Carroll SG, Reddihough DS, et al. Investigation of the timed ‘up & go’ test in children. Dev Med Child Neurol. 2005;47(8):518–524. 16108451 10.1017/s0012162205001027

[7] Carey H, Martin K, Combs-Miller S, et al. Reliability and responsiveness of the timed up and go test in children with cerebral palsy. Pediatr Phys Ther. 2016;28(4):401–408. 27661230 10.1097/PEP.0000000000000301

[8] Dhote SN, Khatri PA, Ganvir SS. Reliability of “Modified timed up and go” test in children with cerebral palsy. J Pediatr Neurosci. 2012;7(2):96–100. 23248683 10.4103/1817-1745.102564 PMC3519092

[9] Katz-Leurer M, Rotem H, Lewitus H, et al. Functional balance tests for children with traumatic brain injury: within-session reliability. Pediatr Phys Ther. 2008;20(3):254–258. 18703963 10.1097/PEP.0b013e3181820dd8

[10] Nicolini-Panisson RDA, Donadio MVF. Normative values for the timed ‘up and go’ test in children and adolescents and validation for individuals with down syndrome. Dev Med Child Neurol. 2014;56(5):490–497. 24206424 10.1111/dmcn.12290

[11] Lei Y, Lam CKY, Lam MHS, et al. Validity and reliability of timed up and go test on dynamic balance in 3-5 years old preschool children. J Yoga Phys Ther. 2017;07(02):266.

[12] Habib Z, Westcott S, Valvano J. Assessment of balance abilities in Pakistani children: a cultural perspective. Pediatr Phys Ther. 1999;11(2):73–82.

[13] Itzkowitz A, Kaplan S, Doyle M, et al. Timed up and go: reference data for children who are school age. Pediatr Phys Ther. 2016;28(2):239–246. 26914719 10.1097/PEP.0000000000000239

[14] Marchese VG, Oriel KN, Fry JA, et al. Development of reference values for the functional mobility assessment. Pediatr Phys Ther. 2012;24(3):224–230. 22735469 10.1097/PEP.0b013e31825c87e7

[15] Kamide N, Takahashi K, Shiba Y. Reference values for the timed up and go test in healthy Japanese elderly people: determination using the methodology of meta-analysis. Geriatr Gerontol Int. 2011;11(4):445–451. 21554510 10.1111/j.1447-0594.2011.00704.x

[16] Kail RV. Children and their development. 6th ed. London (UK): Pearson Education; 2012.

[17] Green SB. How many subjects does it take to do a regression analysis. Multivariate Behav Res. 1991;26(3):499–510. 26776715 10.1207/s15327906mbr2603_7

[18] Ngai SPC, Jones AYM, Jenkins SC. Regression equations to predict 6-minute walk distance in chinese adults aged 55–85 years. Hong Kong Physiotherapy J. 2014;32(2):58–64.

[19] Israel GD. Sampling: the evidence of extension program impact. Program evaluation and organizational development, IFAS [Internet]. Gainesville (FL): University of Florida; 1992 [accessed 18 Dec 2018]. Available from: http://edis.ifas.ufl.edu/pdffiles/PD/PD00500.pdf3/6/2011

[20] Verbecque E, Schepens K, Theré J, et al. The timed up and go test in children: does protocol choice matter? A systematic review. Pediatr Phys Ther. 2019;31(1):22–31. 30557276 10.1097/PEP.0000000000000558

[21] Nicolini-Panisson RDA, Donadio MVF. Timed “up & go” test in children and adolescents. Rev Paul Pediatr. 2013;31(3):377–383. 24142322 10.1590/S0103-05822013000300016 PMC4182976

[22] Koo TK, Li MY. A guideline of selecting and reporting intraclass correlation coefficients for reliability research. J Chirope Med. 2016;15(2):155–163. 10.1016/j.jcm.2016.02.012 PMC4913118 27330520

[23] Craney TA, Surles JG. Model-dependent variance inflation factor cutoff values. Qual Eng. 2002;14(3):391–403.

[24] Butz SM, Sweeney JK, Roberts PL, et al. Relationships among age, gender, anthropometric characteristics, and dynamic balance in children 5 to 12 years old. Pediatr Phys Ther. 2015;27(2):126–133. 25695196 10.1097/PEP.0000000000000128

[25] Nolan L, Grigorenko A, Thorstensson A. Balance control: sex and age differences in 9- to 16-year-olds. Dev Med Child Neurol. 2005;47(7):449–454. 15991864 10.1017/s0012162205000873

[26] Geldhof E, Cardon G, De Bourdeaudhuij I, et al. Static and dynamic standing balance: test-retest reliability and reference values in 9 to 10 year old children. Eur J Pediatr. 2006;165(11):779–786. 16738867 10.1007/s00431-006-0173-5

[27] Mickle K, Munro B, Steele J. Gender and age affect balance performance in primary school-aged children. J Sci Med Sport. 2011;14(3):243–248. 21276751 10.1016/j.jsams.2010.11.002

[28] Kolic J, O’Brien K, Bowles K‐A, et al. Understanding the impact of age, gender, height and body mass index on children’s balance. Acta Paediatr. 2020;109(1):175–182. 31301080 10.1111/apa.14933

[29] Schedler S, Kiss R, Muehlbauer T. Age and sex differences in human balance performance from 6-18 years of age: a systematic review and meta-analysis. PLoS One. 2019;14(4):e0214434. 30964877 10.1371/journal.pone.0214434 PMC6456289

[30] Lemos L, David A, Mota C. Development of postural balance in Brazilian children aged 4-10 years compared to young adults. Rev Bras Cineantropom Desempenho Hum. 2016;18(4):419–428.

[31] Steindl R, Kunz K, Schrott-Fischer A, et al. Effect of age and sex on maturation of sensory systems and balance control. Dev Med Child Neurol. 2006;48(6):477–482. 16700940 10.1017/S0012162206001022

[32] Riach CL, Hayes KC. Maturation of postural sway in young children. Dev Med Child Neurol. 1987;29(5):650–658. 3666328 10.1111/j.1469-8749.1987.tb08507.x

[33] Odenrick P, Sandstedt P. Development of postural sway in the normal child. Hum Neurobiol. 1984;3(4):241–244. 6526710

[34] Hirabayashi S, Iwasaki Y. Developmental perspective of sensory organization on postural control. Brain Dev. 1995;17(2):111–113. 7542846 10.1016/0387-7604(95)00009-z

[35] Kirshenbaum N, Riach C, Starkes J. Non‐linear development of postural control and strategy use in young children: a longitudinal study. Exp Brain Res. 2001;140(4):420–431. 11685395 10.1007/s002210100835

[36] Riach C, Starkes J. Velocity of centre of pressure excursion as an indicator of postural control systems in children. Gait Posture. 1994;2(3):167–172.

[37] Habib Z, Westcott S. Assessment of anthropometric factors on balance tests in children. Pediatr Phys Ther. 1998;10(3):101–109.

[38] Bustam IG, Suriyaamarit D, Boonyong S. Timed up and go test in typically developing children: protocol choice influences the outcome. Gait Posture. 2019;73:258–261. 31382232 10.1016/j.gaitpost.2019.07.382

